# Supplementary figures and images for: Real-time resolution studies of the regulation of lactate production by hexokinases binding to mitochondria in single cells
Source: PLoS One. 2024 Mar 8;19(3):e0300150. doi: 10.1371/journal.pone.0300150 (PMC10923494; doi:10.1371/journal.pone.0300150)

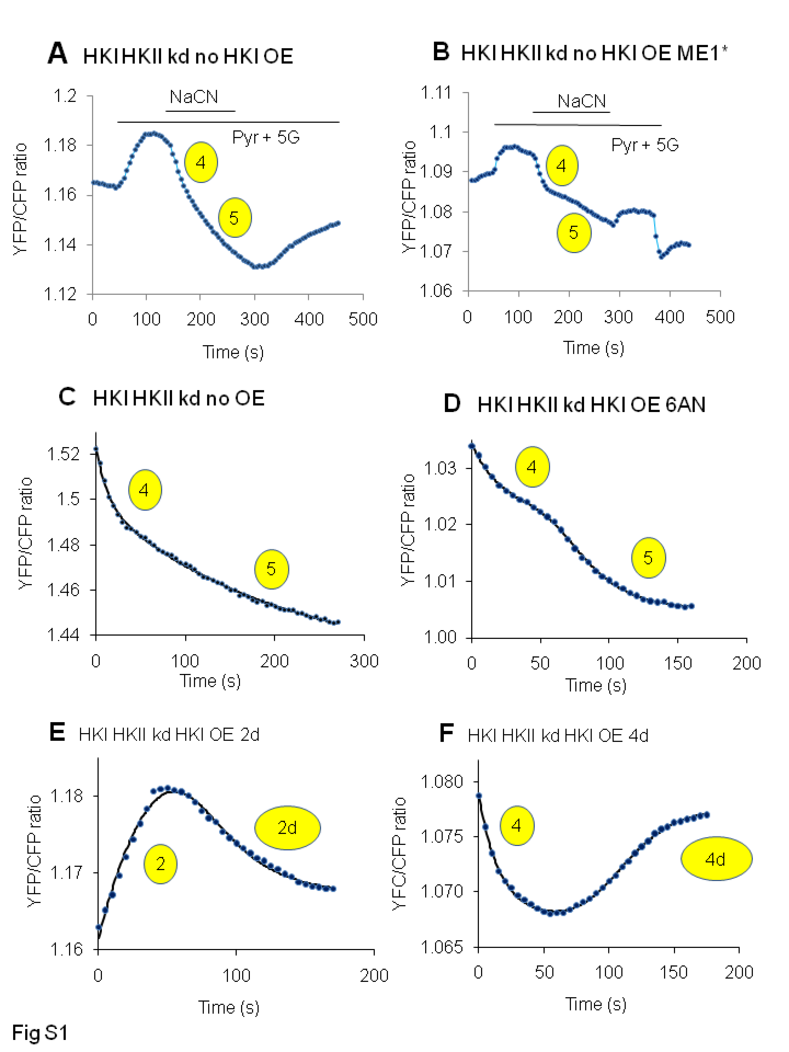

Supplement: S1 Fig — Panels A and B show traces taken from panels A1 and A2 in Fig 4, on an extended time scale, to emphasize the time course of the effect of Pyruvate and NaCN in Hki/HKII Kd cells in the presence and absence of ME1*. Panels C, D, E and F show curve fitting of the changes in FRET ratio. In this figure and all the other figures a downward trend in FRET ratio indicates an increase in intracellular lactate. In panel C the increase in intracellular lactate levels evoked by NaCN (phases 4 and 5) was fitted with a sum of two exponential functions. The amplitudes and time constants of the two phases were derived from the fit. In D the changes evoked by the inhibitor 6AN was best fitted using a combination of exponential and sigmoidal functions. In this case the amplitude of phase 5 was derived from the difference between the calculated maximum and minimum. In E and F the effects of NaCN on the amplitude of phases 2 and 4 was estimated as the difference between the beginning and end of the fitted traces. When the value at end of the trace exceeded that at the beginning a negative value for the amplitude was derived from the calculation. (TIF) [file pone.0300150.s001.tif]

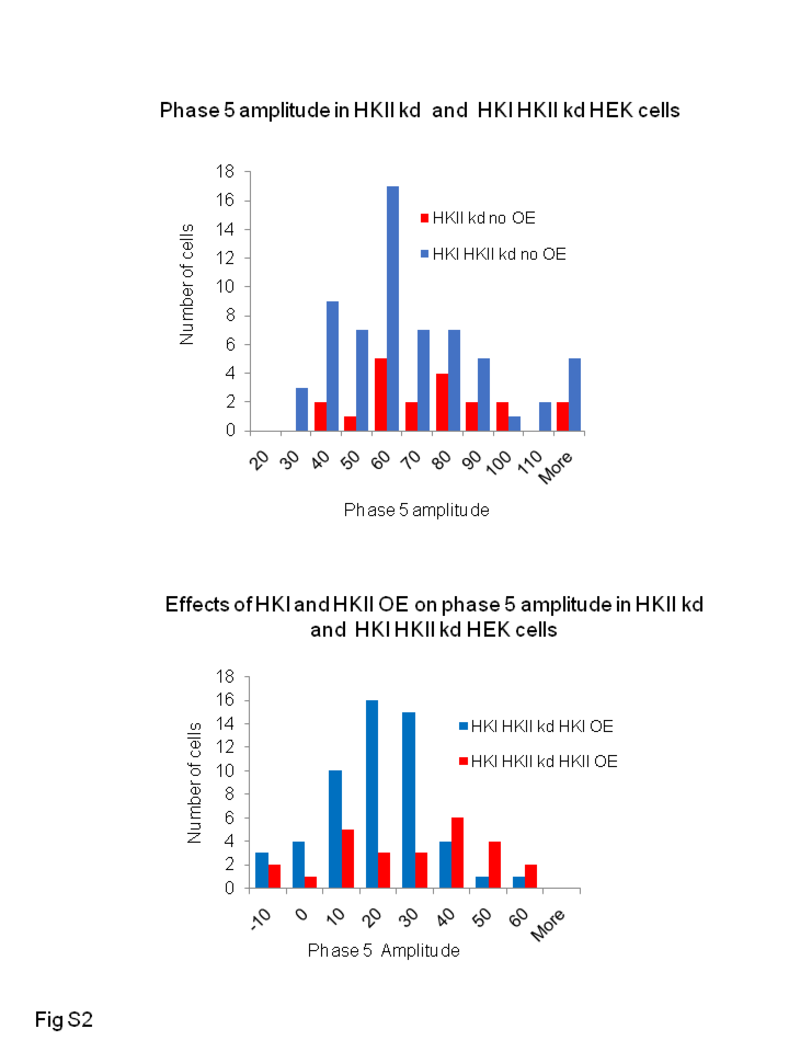

Supplement: S2 Fig — The upper panel depicts the effects of knocking down (kd) HKII and HKI HKII in HEK cells. In HKI HKII kd (no OE) Mean phase 5 normalized amplitude was 64.4 ± 3.55 (n = 63). In HKII kd (no OE) Mean phase 5 normalized amplitude was 71.6 ± 5.08 (n = 21). F-test yielded a P value of 0.12. A t-test (equal variance) P value was 0.97. The shapiro-wilk test P value for HKI HKII kd no OE was 0.1, and 0.07 for HKII. The lower panel depicts the effects of the overexpression of HKI and of HKII in HEK cells in which HKI and HKII had been previously knocked down. In HKI HKII kd cells, HKI OE Mean phase 5 normalized amplitude was 16±1.8 (n = 55). In HKI HKII kd cells, HKII OE Mean phase 5 normalized amplitude was 23.4±3.8 (n = 27). An F-test yielded a P value of 0.012 and a t-test (unequal variance) yielded P<0.0005. The shapiro-wilk test P value for HKI OE was 0.07, and 0.9 for HKII OE. (TIF) [file pone.0300150.s002.tif]

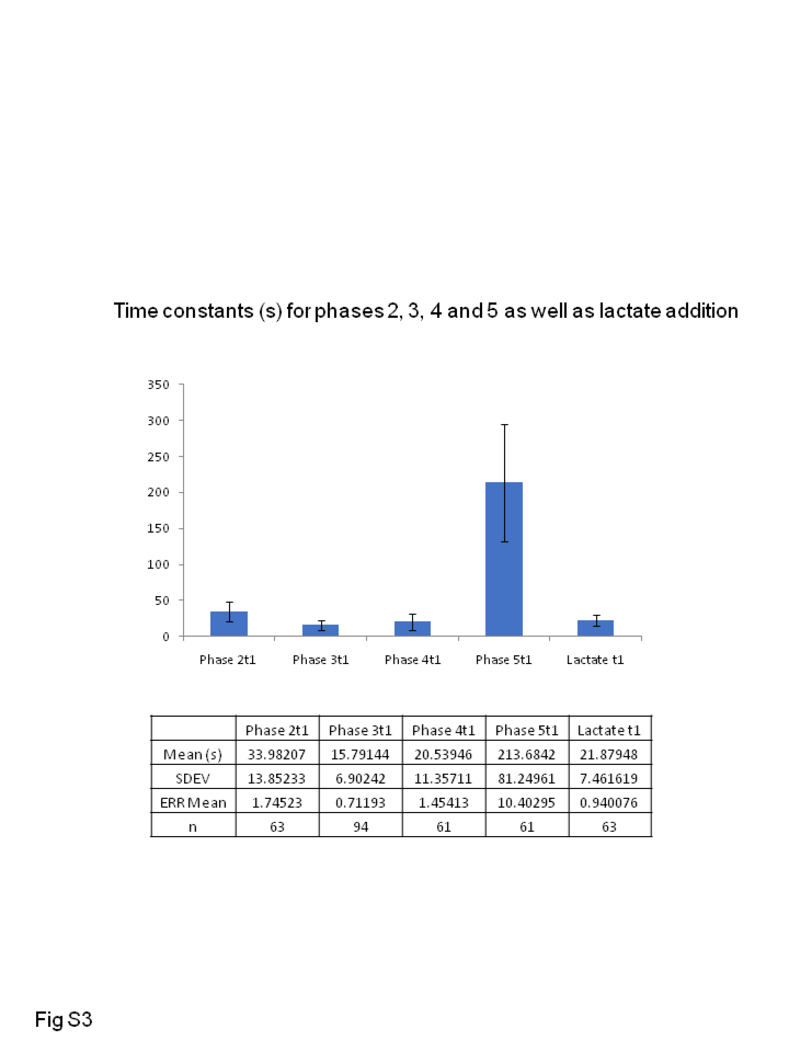

Supplement: S3 Fig — Phase 2t1 is for the addition of pyruvate, phase 3t1 for the addition of AOA, Phases 4t1 and 5t1 for the addition of NaCN and lactate t1 for the addition of lactate. Mean 33.98207 15.79144 20.53946 213.6842 21.87948. SDEV 13.85233 6.90242 11.35711 81.24961 7.461619. ERR Mean 1.74523 0.71193 1.45413 10.40295 0.940076. (TIF) [file pone.0300150.s003.tif]

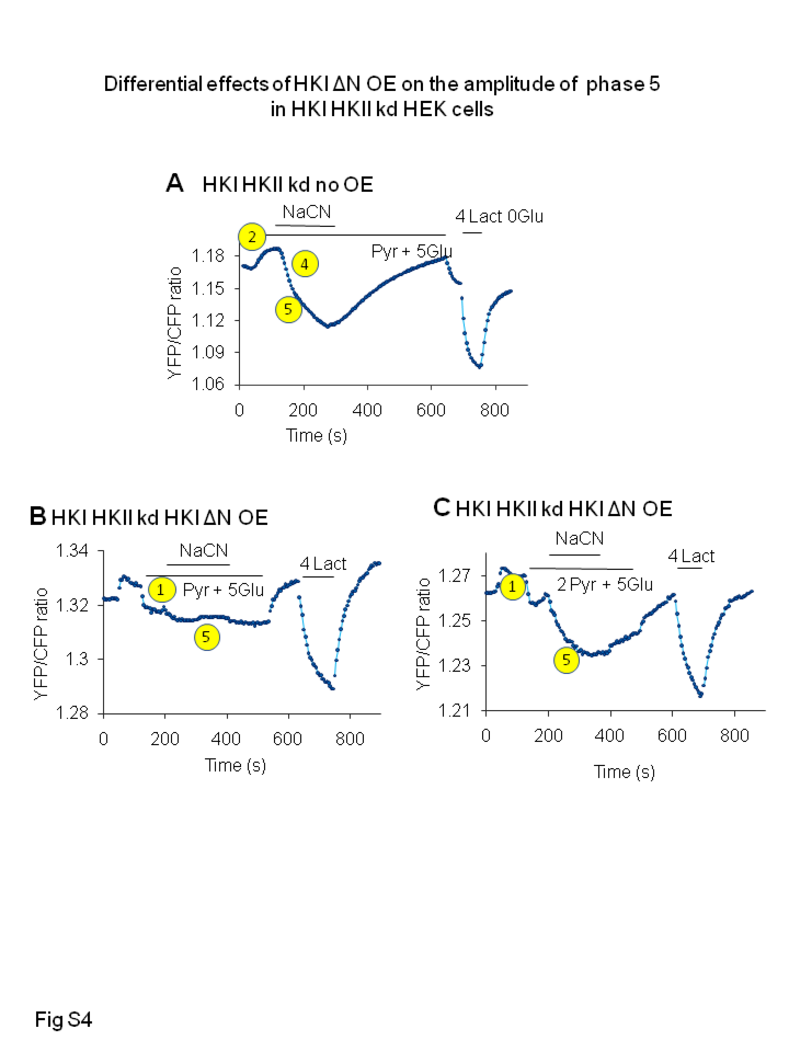

Supplement: S4 Fig — Data in panels A shows the effects of NaCN addition in HKI HKII kd cells for reference. Clear Phases 2, 4 and 5 are observed in this case. Panels B and C show two very different results observed with ΔN HKI overexpression (OE). In one case (panel B) overexpression of the mutant HKI had effects very similar to those of the wild type with only a phase 1 and no phases 4 and 5. However, in other cells a clear phase 5 was observed (Panel C). Thus, in this case overexpression of the ΔN HKI did not block the increase in lactate evoked by NaCN. This dichotomy is consistent with the imaging data obtained with ΔN HKI-YFP (Fig 6). (TIF) [file pone.0300150.s004.tif]
